# The Non-Panel Movement

**Published:** 1913-10-18

**Authors:** 


### THE NON-PANEL MOVEMENT.

The article we publish elsewhere on anti-panel and panel practice under the Insurance Act may be studied with advantage by every practitioner of medicine in the United Kingdom. The upheaval which has taken place in the profession as a first result of the coming into force of the National Insurance Acts would have been infinitely greater had it not been for the wise restraint exercised by the managers of our voluntary hospitals throughout the country. It is an incontrovertible fact, known to the actuaries and to all who are intimate with the inner working of National Insurance and the plan upon which it rests, that the voluntary hospitals are at present bearing the whole cost of treating all the cases of serious illness and disablement which occur amongst the insured. It is a further fact that unless the voluntary hospitals were defraying the heavy cost of this most costly work the British system of National Insurance must have broken down financially and landed its authors in ruinous discredit. We hope that these patent facts will be clearly understood by members of the medical profession whose interests they are likely to affect seriously when the first valuation is made in 1916.

The position to-day is, roughly, that the panel practitioner appears to have fallen on good times, for he is able to reap immediate cash benefits from National Insurance and at the same time to make serious inroads on the practice of the non-panellers. Yet it must be remembered that the non-panellers are the men who had the grit to stand by their word, and to this resolute action on their part the medical profession is enormously indebted, for without it the practice of medicine would be so discredited in public estimation as to fall from the status of a learned profession to that of a mere trade. It follows that it is of the first importance that the non-panellers, who are at present ranged in sections under various titles up and down the country, should co-operate together and form themselves into one great organisation for their mutual protection and in defence of the highest principles of medical practice.

The next few weeks are likely to prove of momentous importance to the best interests of the medical profession. Steps are being taken to unite the non-panellers, and the National Medical Union has intimated its willingness to join hands with other non-panel centres with the object of linking up all non-panel bodies. The principle upon which this united action rests is one of complete independence for non-panellers and an insistence that they shall control and manage their own affairs independently. We are glad to have an intimation that there is a general feeling amongst the heads of the existing non-panel organisations that the first consideration is to secure united and combined action. The non-panel movement has therefore taken on new strength and force, and we believe it will be found that by wise counsel and mutual conciliation all differences will be sunk in a united effort to strengthen the non-panel movement in every possible way. Abundant evidence has been submitted to us in proof of the practically unanimous view of the non-panellers that they must consolidate and unite their forces. All danger of divided counsels, or a split, promise to be removed by the self-denying and statesmanlike action of the leaders of the various sections who, being first of all non-panellers to the core, will work unitedly in support of one central organisation.

